# Influence of beech and spruce on potentially toxic elements-related health risk of edible mushrooms growing on unpolluted forest soils

**DOI:** 10.1038/s41598-022-09400-9

**Published:** 2022-03-30

**Authors:** Václav Pecina, Martin Valtera, Karel Drápela, Radek Novotný, Petr Vahalík, Renata Komendová, Martin Brtnický, David Juřička

**Affiliations:** 1 Institute of Chemistry and Technology of Environmental Protection, Faculty of Chemistry, Brno University of Technology, Purkyňova 118, 61200 Brno, Czech Republic; 2 Department of Agrochemistry, Soil Science, Microbiology and Plant Nutrition, Faculty of AgriSciences, Mendel University in Brno, Zemědělská 1, 613 00 Brno, Czech Republic; 3 Department of Geology and Soil Science, Faculty of Forestry and Wood Technology, Mendel University in Brno, Zemědělská 3, 613 00 Brno, Czech Republic; 4 Department of Forest Management and Applied Geoinformatics, Faculty of Forestry and Wood Technology, Mendel University in Brno, Zemědělská 3, 613 00 Brno, Czech Republic; 5 Forestry and Game Management Research Institute, Strnady 136, 252 02 Jíloviště, Czech Republic

**Keywords:** Biogeochemistry, Environmental sciences

## Abstract

Atmospheric deposition-related potentially toxic elements (PTEs) can contaminate mountain forest ecosystems. The influence of tree species is being increasingly recognised as an important factor in the deposition loads in forest soils. However, relevant modelling studies about the forest pollution with PTEs, concerning the tree species composition, are lacking. The aim of this study was to evaluate the effect of European beech (*Fagus sylvatica* L.) and Norway spruce (*Picea abies* (L.) H. Karst.) on soil and mushroom pollution and the associated health risks to define their significance for pollution modelling. Therefore, topsoil samples and samples of eight edible mushroom species were taken from 51 mature beech- and spruce-dominated stands. The results showed that forest composition had an indirect influence on the PTEs contents in the topsoil; it significantly differentiated the relationship between PTEs and soil C as the beech stands showed significantly increasing PTEs content with increasing C content. Despite the absence of soil pollution, above-limit levels of Cd and Zn were found in mushrooms. The total content of PTEs in mushrooms posed a potential health risk to consumers in 82% of the samples. The most Cd-contaminated and potentially the riskiest species for consumption was *Xerocomellus pruinatus* (Fr. and Hök) Šutara. The results suggest that the source of PTEs for mushrooms is not only the soil but probably also the current wet deposition. The influence of the forest type on the accumulation of PTEs in mushrooms was confirmed mainly due to the strongly divergent behaviour of Zn in beech- vs. spruce-dominated stands. The results point to the need to evaluate mushroom contamination even in the contamination-unburdened forest areas. For future modelling of PTEs pollution in forests, it is necessary to differentiate the tree species composition.

## Introduction

Environmental pollution with potentially toxic elements (PTEs) is a global environmental problem. Studies addressing this issue focus mainly on urban, mining, or agricultural areas due to the higher risk of direct human exposure and potentially easier intoxication. Less attention is paid to forest ecosystems^1^, which are expected to be less polluted due to the predominant absence of nearby contamination sources. However, recent results suggest that these assumptions may be wrong^2,3^.

Intensive industrialisation began in Central Europe in the eighteenth century and the resulting environmental pollution led to strong impacts on forest ecosystems, which are ongoing^4–6^. PTEs can be transported by air far from their source such as industrial areas. The depositions are intercepted by forest vegetation, precipitated to the forest floor, and subsequently, infiltrated into the mineral soil with percolating water^1,3^. Due to the affinity of PTEs to soil organic matter and their long-term persistence in soil^7^, the current inputs as well as the historical accumulation of PTEs are important when determining the critical loads^8^.

Regular high intake of PTEs poses a health risk to all living organisms^7,9^. Mushrooms are significant accumulators of PTEs from the surrounding environment and therefore pose a potential risk to their consumers^10–13^. The accumulation of PTEs in fruiting bodies of wild ectomycorrhizal mushrooms with culinary uses depends on the ability of fungal hyphae to absorb them from the soil and transport them through the mycelium^14^. These mushroom species preferentially absorb nutrients and other substances from the forest floor and mineral topsoil^10,15^, i.e. from potentially the most contaminated soil layers^16^. Mushroom species are generally highly discriminating regarding the accumulation of PTEs^17^. However, Cd, Zn, and Cu can be effectively accumulated in many mushroom species^14,18^. In some countries, mushroom picking is a kind of national hobby, involving up to 65% of households in the Czech Republic, for example^19^. Therefore, the risk of large-scale intoxication by contaminated wild-growing edible mushrooms can be high.

The tree species composition of a forest ecosystem can be significant for the overall contamination load^16,20^. There is a difference in the throughfall deposition of elements in coniferous stands, which can capture more dust and gaseous pollution, compared to deciduous species due to the higher total surface area of the needles and the evergreen nature of the conifers. The depositions are washed by precipitation into the soil, which significantly affects topsoil properties^20–23^. However, there is also the view that evergreen conifers can store contaminated dust in their foliage for a few seasons, while deciduous trees transfer the annual input of dust with PTEs into the soil as litterfall. In addition, the stem flow of deciduous trees generates higher solute fluxes to the soil in comparison to solute fluxes during throughfall^24^. Norway spruce (*Picea abies* (L.) H. Karst.) and European beech (*Fagus sylvatica* L.) are given special attention as they are two of the most economically important European tree species^25^. Spruce has a higher potential for capturing atmospheric pollutants than beech as it is associated with higher S and N depositions^22^.

Despite ongoing research, there are uncertainties and therefore, relevant studies and methodologies for modelling tree species-related forest pollution with the risk of picking contaminated mushrooms are lacking. The topic is mostly studied separately with a strong local focus on one or two selected stands as a subtopic to evaluate the impact of tree species on soils^6,24^ or the risk of consuming mushrooms from areas with proven heavily polluted soils^14,26^. Data from mountain areas without a significant local large-scale source of pollution are mostly lacking.

The objectives of this study were (1) to assess topsoil pollution with PTEs (Cd, Cu, Pb, and Zn as potentially serious contaminants related to atmospheric deposition) in the forests of the mountain region; (2) to assess the contamination of mushrooms and the potential health risks associated with their consumption; (3) to evaluate the effect of beech and spruce on PTE contents and risks; and (4) to identify the main factors for spatial modelling of PTE distribution in forest topsoil. Tree species (forest type) are assumed to be a significant factor in spatial modelling of PTE pollution in forest areas. Particularly, we hypothesise that (H1) despite the absence of a significant local pollution source, the topsoil in the studied stands will be polluted due to atmospheric depositions from remote industrial areas and (H2) due to the expected higher topsoil pH, there will be a higher topsoil accumulation of PTEs in beech stands. On the other hand, for spruce stands, we hypothesise that (H3) due to the higher spruce acidy-related mobility of PTEs, there will be significantly higher contents of PTEs in mushrooms and related potential health risks.

## Materials and methods

### Study area

**Figure 1:**
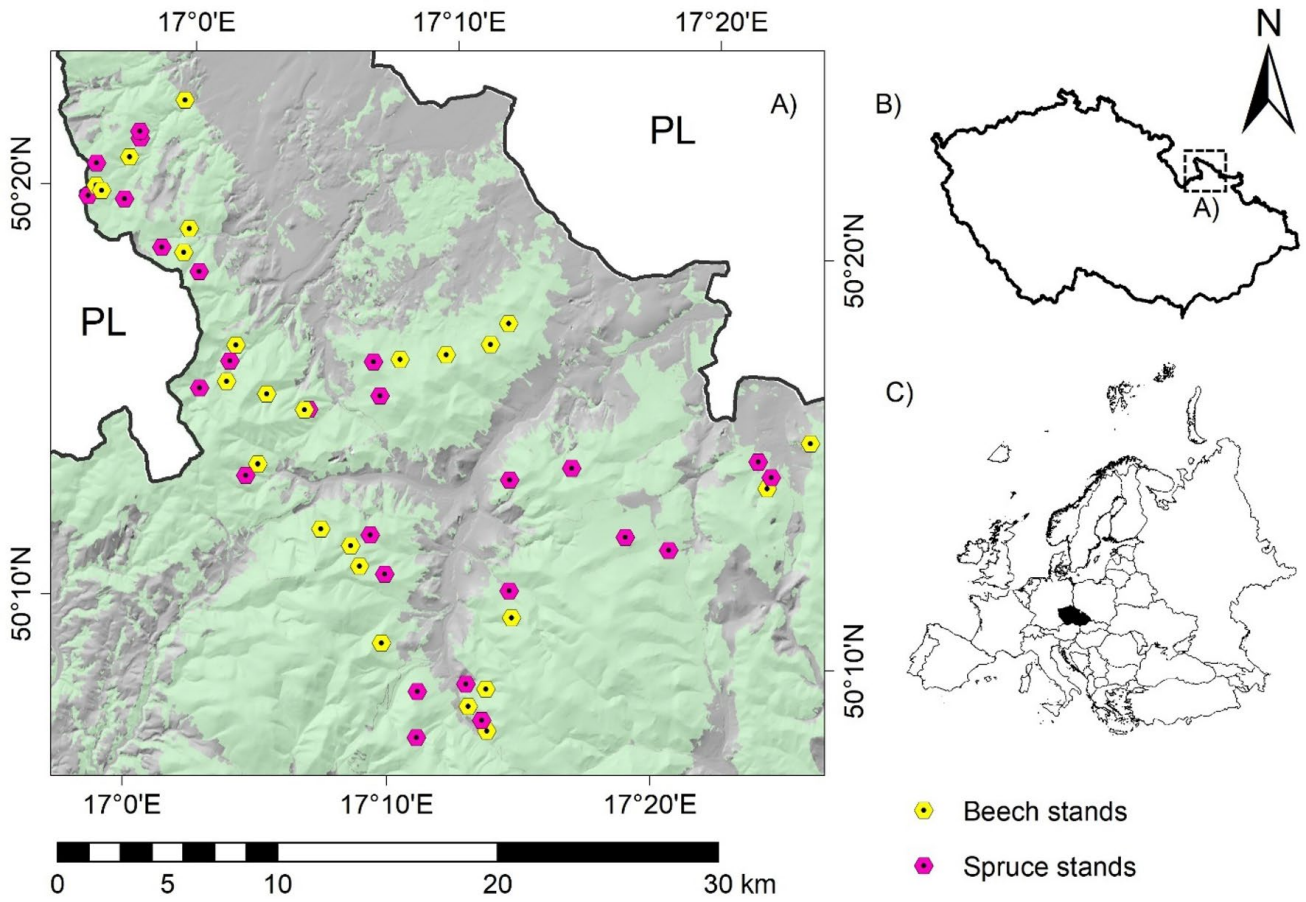
Study area with the sampling sites at the background layer, derived from the regional forest-development plans (forest areas are green) (**A**); digital model of the Czech Republic was used for shading; PL = Poland; location of the study area within the Czech Republic (**B**) and Europe (**C**).

The study was carried out in a highly tourism-exposed region of the Jeseníky Mountains, the Czech Republic (Fig. 1). The region is marginally affected by historical and current contamination originating from atmospheric depositions, mostly from the industrial Upper Silesian Coal Basin^27,28^. The studied area was located at altitudes of 396–990 m, with an average annual precipitation of 810 mm (predominance in the growing season) and an average annual temperature of 7.8 °C (considered from 1989 through 2019)^29^. Forest stands in the area are predominantly even-aged Norway spruce (*Picea abies* (L.) H. Karst.) and European beech (*Fagus sylvatica* L.) monocultures growing on Cambisols and Podzols. The atmospheric deposition data (in a square network of 1 × 1 km) are available only for Cd and Pb, with a range of 0.3–0.6 mg/l and 5.0–8.2 mg/l, respectively^28^. Further information on the study area is provided in chapter 2.2.

### Soil and mushroom sampling

Beech- and spruce-dominated (considered for > 80% basal area of the species) stands were selected for soil and mushroom sampling according to the following parameters: (a) area > 1 ha; (b) age 80–100 years; (c) absence of a local source of pollution (e.g. built-up area, heaps, roads, and landfills); and (d) comparable bedrock consisting of acid metamorphic rocks (mostly phyllites and gneiss). According to the parameters, 51 stands (26 × spruce and 25 × beech; Fig. 1) were selected after field verification. The methodology partly followed previous studies^16,30^. A random plot was sampled within each of the stands, which included some previously published soil data (6 × spruce and 6 × beech stands^30^). To avoid edge effects, the plots were placed at least 25 m apart from the stand edge. At each plot, a standard composite sample was collected from the 0–2 cm mineral soil using a plastic shovel at three sampling points spaced 4–6 m apart, so the sampling was never performed twice under the canopy projection of a single tree.

Mushroom samples of the species from the *Boletaceae* family commonly used for culinary purposes, were collected from each stand, depending on the current occurrence of the fruiting bodies of the species. The fruiting mushroom bodies were sorted into paper bags as a mixed sample for each mushroom species. To minimise the bias due to the presence of other tree species in the stands, all mushroom samples were collected only under the dominant tree species. A total of 96 mushroom samples of eight species were collected (Table S1).

### Sample preparation and analysis

The soil samples were dried at room temperature, homogenised, and treated to fine soil following the standard ČSN ISO 11464.

The pH was measured in a suspension of mineral and organic soil in water, with a soil-to-water volume ratio of 1:5. Total C contents of oven-dry samples were determined using a catalytic tube combustion method with thermal conductivity detector on an elemental analyser VarioMAX CNS (Elementar Analysensysteme GmbH, Germany) following ČSN ISO 10694. Total C content was analysed to express the organic matter content in soil as they are approximately equal.

The fruiting bodies of the mushrooms were manually cleaned of the coarse debris (parts of plants, forest floor, or mineral particles) with a ceramic knife. The samples were sliced and dried at room temperature for several weeks in a dust-free environment. After drying, the mushroom samples were homogenised by grinding to powder in a stainless-steel mill for subsequent PTEs content analysis.

Pre-prepared soil and mushroom samples were digested in *aqua regia* (mixture of HNO_3_ and HCl in a ratio of 1:3) to determine the content of Cd, Cu, Pb, and Zn. The digestion took place in a microwave digestion platform ETHOS EASY (Milestone, Italy). After digestion, the samples were quantitatively transferred with Milli-Q water (Merck, Germany) to a volume of 25 ml. The concentration of the elements in the digested samples was measured on an atomic absorption spectrophotometer ContrAA 800D (Analytik Jena, Germany) with a continuous radiation source (Xe lamp), a high-resolution monochromator, and a dual flame system (FAAS) or electrothermal atomiser (ET-AAS). The optical system is based on a high-resolution Echelle monochromator and a sensitive CCD detector. Cd, Cu, and Pb were determined using the ET-AAS method, while Zn was determined using the FAAS method. Blanks, triplicate measurements, and matrix reference material measurements (METRANAL 31, METRANAL 33, and METRANAL 34; Analytika, Czech Republic) were performed for quality assurance and quality control (Table S2). The range of relative standard deviations of the triplicate measurements was 0.5–6.8% for soils and 0.1–8.3% for mushrooms. Recoveries related to the reference materials ranged between 93.5 and 112.3% (METRANAL 31), 94.2 and 103.6% (METRANAL 33), and from 96.3 to 102.9% (METRANAL 34). The limits of detection for Cd, Cu, Pb, and Zn were 0.003, 0.012, 0.033, and 0.007 mg/kg, respectively. Mushroom results were given for dry weight.

### Soil pollution assessment

The Integrated Nemerow Pollution Index (IPI_N_) was used for the assessment of the PTEs-related soil pollution level using two different standards (see the following text). IPI_N_ classes are safe (≤ 0.7), precaution (0.7–1), slight pollution (1–2), moderate pollution (2–3), and heavy pollution (≥ 3). IPI_N_ was calculated as follows^31^:

$${\mathrm{PI}}_{\mathrm{i}}= \frac{{\mathrm{C}}_{\mathrm{i}}}{{\mathrm{L}}_{\mathrm{i}}}$$

$${\mathrm{IPI}}_{\mathrm{N}} = [(\mathrm{P}{{\mathrm{I}}^{2}}_{\mathrm{avg}} + {{\mathrm{PI}}^{2}}_{\mathrm{max}})/2{]}^{1/2}$$

where PI_i_ means single pollution index of individual PTE, C_i_ means the content of the PTE, L_i_ means the limit value of the PTE—Dutch Target Value (VROM^32^) or Czech legislation (Decree No. 153/2016 Coll.^33^), PI_avg_ means the average value of all PI_i_ of the PTEs, and PI_max_ means the maximum PI_i_ value of the PTEs.

### Mushroom contamination and health risk assessment

The contents of PTEs in mushrooms were compared with the Czech legislative limits from Decree No. 298/1997 Coll.^34^ for Cu and Zn and Decree No. 53/2002 Coll.^35^ for Cd and Pb (the last one available; currently, there is no legislative limit for wild-growing mushrooms in the Czech Republic) and the usually reported PTE contents for species grown in unpolluted sites^18^ in the assessment of a contamination.

To evaluate the relationship between the mushroom and soil PTE contents, the ability to accumulate a PTE was measured using Bioconcentration Factor (BCF)—a simple method to quantitatively characterise the transfer of available PTEs from the soil to the mushroom. BCF is calculated as the ratio of the mushroom PTE content to the total soil PTE content. The stand-specific soil values were used for the calculation, i.e., the PTE value in the mushroom sample was divided by the respective PTE value in the soil from the stand where the mushroom was collected. Mushrooms with a BCF value of < 1 correspond to PTE excluders while a BCF value of > 1 to accumulators^11,36^.

The potential health risk of the mushrooms, expressed as the Health Risk Index (HRI), was calculated as the ratio of daily intake of metal (DIM) through the edible mushrooms to the oral reference dose (RfD_o_)^37,38^. HRI values > 1 represent a potential health risk. RfD_o_ values used for Cd, Cu, Pb, and Zn were 0.001, 0.04, 0.0035, and 0.3 mg/kg/day, respectively^31,39^. Daily intake was calculated using the following equation^26,37,38,40^:

$${\mathrm{DIM}}_{\mathrm{i}}=\frac{{\mathrm{C}}_{mushroom}\times {\mathrm{D}}_{IM}}{\mathrm{BW}}$$

where C_mushroom_ represents the PTE content in the mushrooms (mg/kg, based on dry weight); D_IM_ represents the daily intake of mushrooms (0.03 kg)^13,38^; and BW represents an average bodyweight of the consumer (74.6, an average of men and women in the Czech Republic)^41^.

### Statistical and spatial analysis

Statistical analysis of data sets was performed in the Statistica 12 program, both for the whole sets and subsets of spruce and beech stands separately. Due to the lack of mushroom biomass for analysis after drying from one spruce stand, only 50 stands were statistically analysed. In addition to the standard descriptive statistics, normality tests (Shapiro–Wilk) were performed, and the basic graphical methods of exploratory analysis were used (box plot, QQ plot and histogram). Since most of the data sets did not show a normal distribution and a larger number of outliers were found, subsequent comparisons of the beech and spruce stands were performed using a nonparametric test (Mann–Whitney U test). Linear regression analysis was used to determine the relationship between PTEs’ contents and soil properties. The tests were evaluated at the level of significance *p* = 0.05.

Spatial distribution maps of PTEs were created in the program ArcGIS 10.4. The position of the sampling sites and the values of Cd, Cu, Pb, and Zn contents in the soil and mushrooms were converted to a geodatabase format. Spatial interpolation of the soil PTE contents was performed by the Geographically Weighted Regression (GWR) method. The dependent, i.e. modelled variable of GWR, was the soil PTE content, and the independent variable was the total C content as a continuous variable significantly influencing the PTEs in the soils. Due to the different density and irregular spatial distribution of sites with variable neighbour distance, the context of the positions was analysed using the adaptive kernel of weighted regression. The scope of the kernel of weighted regression used Akaike's information criterion for small selections (AICc). The spatial output of the GWR was subsequently processed by the method of maximum probability classification, where the rasters of beech and spruce representation in forest stands were used as independent variables. The weight of the independent variable was the percentage share of beech or spruce in the stands compared to other tree species. Due to the high variability in mushroom contamination (both within and between mushroom species), a point presentation of the results was performed with an indication of potential health risk (HRI).

## Results and discussion

### Soil pollution and tree species (forest type) influence assessment

The overall IPI_N_ assessment classified the soils as safe according to both calculation variants (Table S3). Despite long-term atmospheric deposition of PTEs^28^, originating predominantly from the heavy-industry areas of Silesia^27,42^, the severity of the accumulated soil contamination does not pose any risk in the context of the applied standards^32,33^. Although other studies^3^ have pointed to the potential risk of a serious forest soil enrichment with PTEs even in the case of remote protected areas, the hypothesis H1 was not confirmed.

**Figure 2:**
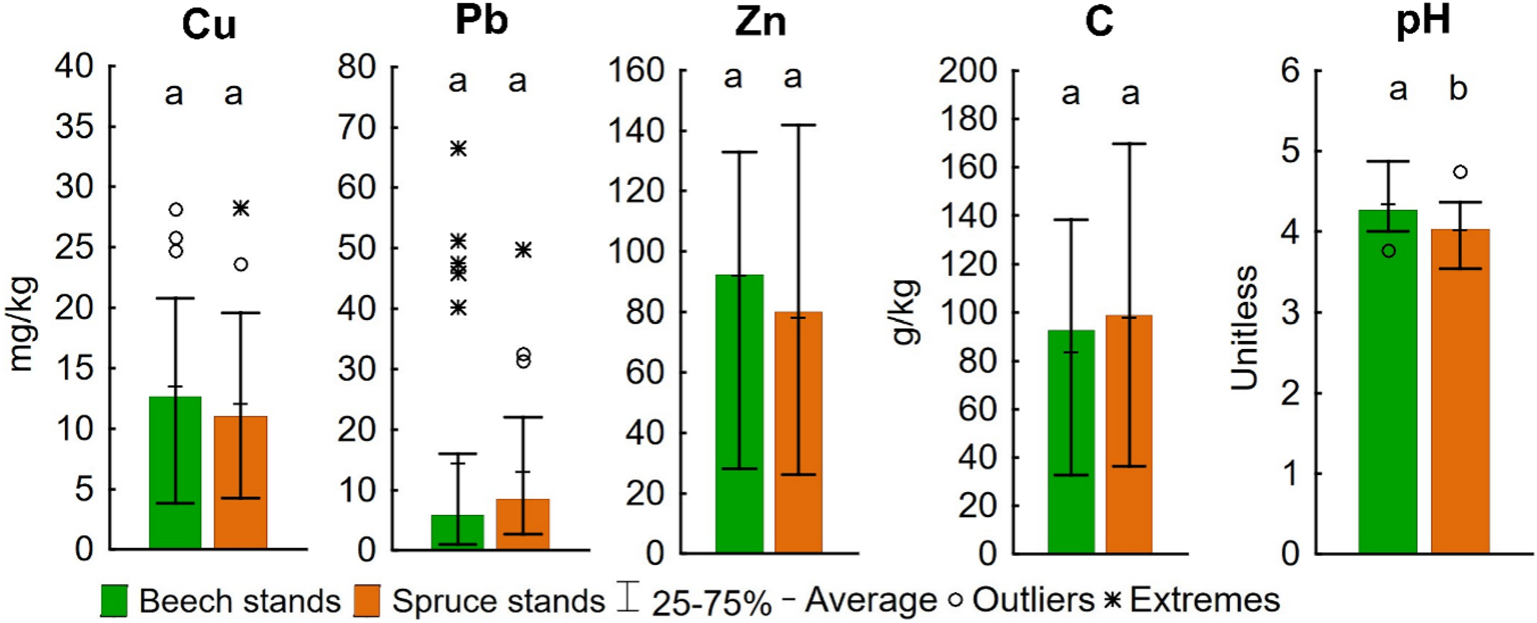
Medians of PTEs contents, pH and C contents in beech and spruce stands soils; different lowercase letters indicate significant differences between forest types (Mann–Whitney U test, *p* = 0.05).

Minor differences between beech and spruce stands were indicated in the IPI_N_ values (Table S3). Similarly, there were no significant differences in the soil contents of Cu, Pb, and Zn (Fig. 2). The cadmium content (Table S3, S4) was predominantly below the limit of detection; therefore, it was omitted from the statistical comparison and graphical presentation. Consequently, at the larger scale, the hypothesis H2 should be rejected.

The absence of differences between the species-related soil pollution is in contradiction to the observed increased magnetic susceptibility of topsoil in the beech as compared to the spruce stands^24^. Similarly, Novotný et al.^30^ observed significantly higher topsoil (0–2 cm) contents of Pb and Zn in beech as compared to spruce; the authors explained this phenomenon by the different effects of the tree species on soil chemistry and the presumed differences in PTEs’ mobility, including their retention and redistribution within the soil profile^30^. A probable reason for the difference compared to the literature is that the results of both studies are based on a smaller number of sites (2 and 12, respectively). Because a larger sampling range here (51 sites), specific site factors could outweigh species factors despite the precise methodology in the selection of the stands and characteristically similar defined region. The assumption is that the predetermining site factor was the slightly different atmospheric depositions resulting from the spatial distribution of individual forest stands (see chapter 2.1).

Soil pH and the quantity and quality of soil organic matter are essential for PTEs cycling and stabilisation in soils^3,9,43^. Spruce-related soil acidification^20,22,44^ resulted in a significantly lower topsoil pH in the spruce as compared to the beech stands (Fig. 2). This could initiate a higher mobility of PTEs and their bioavailability for plants and other living organisms^42,43^. Although species effect alone seems to have a small to no relevance for topsoil enrichment with PTEs, an indirect influence of the tree species on topsoil PTEs’ contents was demonstrated in the correlations of Cu, Pb, and Zn contents to the other soil properties (Table 1). The overall medium to strong positive correlations of Cu, Pb, and Zn with C in the beech-dominated stands confirmed the important role of soil organic matter quantity and quality in the PTEs’ accumulation in soil. In contrast, there were no significant relationships of Cu, Pb, or Zn with C and pH in the spruce-dominated stands. Therefore, it was necessary to reconsider the forest type factor for spatial modelling of soil pollution with PTEs (Figure S1).

**Table 1:** Linear regression models of soil properties and PTEs contents for beech and spruce stands and for the whole dataset; bold values indicate strong positive correlations (*r* > 0.5) that were significant at *p* = 0.01.

Dependent	Explanatory	Beech stands			Spruce stands			All data		
Variable	Variable	*p*-value	*r*	*r*^*2*^	*p*-value	*r*	*r*^*2*^	*p*-value	*r*	*r*^*2*^
Cu	pH	0.9731	−0.01	0.0001	0.9475	−0.01	0.0002	0.6991	0.06	0.0031
	C	**0.0095**	**0.51**	**0.2583**	0.4320	0.16	0.0259	0.0340	0.30	0.0885
Pb	pH	0.0819	−0.35	0.1258	0.5587	−0.12	0.0144	0.1559	−0.20	0.0407
	C	**0.0023**	**0.58**	**0.339**	0.1067	0.32	0.1048	0.0016	0.43	0.1854
Zn	pH	0.5759	−0.12	0.0138	0.549	0.12	0.0152	0.4600	0.11	0.0112
	C	**0.0015**	**0.60**	**0.3617**	0.5717	−0.12	0.0135	0.2606	0.16	0.0258

### Mushroom contamination assessment

Despite the absence of soil pollution, the PTE contents in mushrooms (Table S5) exceeded the pollution and hygienic limits (Table 2). Compared to the PTE values for unpolluted sites^18^, contamination of mushrooms is evident, especially by Cd and Zn. The Czech hygienic limits were exceeded in the case of Pb, Cd, and Zn in 2%, 66%, and 100% of the samples, respectively. Furthermore, a report by the Institute of Agricultural Economics and Information^45^ indicated a serious risk of mushroom contamination with Cd in forest ecosystems in the Czech Republic, where 52% of the samples (*n* = 23) were above the hygienic limit of 2 mg/kg. In this study, Cd and Zn contents often reached the values typical for heavily polluted areas^10,13^. This reveals that mushrooms can be heavily polluted even in forests of a mountain region with neither significant close-source contamination nor recognisable soil pollution.

**Table 2:** Average contents of PTEs (mg/kg) in the studied mushrooms compared to the selected limits; bold values are above both limits.

Species	*n*	Cd			Cu			Pb			Zn		
		Avg	*	SD	Avg	*	SD	Avg	*	SD	Avg	*	SD
*Imleria badia*	27	2.59	b	2.52	29.7	ab	9.45	0.60	c	1.01	**220**	ab	57.4
*Boletus edulis*	23	**6.43**	ac	9.28	32.9	a	10.7	1.14	ab	0.82	**269**	a	64.2
*Xerocomellus chrysenteron*	21	**5.68**	abc	4.86	21.4	b	6.80	3.23	a	2.93	157	b	65.8
*Neoboletus luridiformis*	11	3.04	bc	2.53	29.4	abc	8.51	0.90	abc	0.73	**302**	a	84.1
*Xerocomellus pruinatus*	8	**12.1**	a	8.67	26.8	abc	10.9	4.42	a	3.74	145	b	46.8
*Xerocomus subtomentosus*	4	1.82	abc	0.30	11.8	c	6.51	0.30	bc	0.32	184	ab	20.3
Unpolluted sites^I^	–	1–5			20–100			< 5			25–200		
Hygienic limit^II^	–	2.0			80			10			80		

*Different lower-case letters indicate significant differences among species in PTEs contents. SD are standard deviations.

^I^Range of usual PTE contents reported for the mushroom species at unpolluted sites^18^.

^II^Czech legislative limit (Decree No. 298/1997 Coll.^34^ and Decree No. 53/2002 Coll.^35^).

The most Cd-contaminated species was *Xerocomellus pruinatus*, whose average contents exceeded the hygienic limit by more than 6 times (Table 2). In addition, *Boletus edulis* and *Xerocomellus chrysenteron* can be considered as heavily Cd-contaminated. In the case of Zn, *Porphyrellus porphyrosporus*, *Neoboletus luridiformis*, *Boletus edulis,* and *Imleria badia* reached high over-limit values in particular (Table 2, Table S5). Pb and Cu are unlikely to be serious mushroom contaminants, as they had predominantly below-limit values in all the species.

The high variability of the contents of PTEs among the species and at the species level (Table 2 and S3) is common for mushrooms^12,46^ and can be attributed to environmental factors (influence of site and atmospheric depositions) and age and size of fruiting bodies in the sample or age of mycelium^10,26,46,47^.

Due to the common abundance of *I. badia*, *X. chrysenteron,* and *B. edulis*, the greatest research focus is typically on these species. Kalač et al.^13^ found even 2 times higher Cd contents in *B. edulis* (15.2 mg/kg) at sites heavily polluted by the lead smelter, as compared to the other two species. Strong accumulation of Cd in *B. edulis*, as compared to the other species (Table 2), was also found by Cocchi et al.^17^ and Komárek et al.^10^. The results of this study were also consistent with the findings of Komárek et al.^10^ in the accumulation of Cu and Zn in *B. edulis*, as compared to *X. chrysenterone* (Table 2). Also, Alonso et al.^47^ found *B. edulis* to be the most important Cu accumulator of these three species, while the strongest accumulation of Zn found in *I. badia*. Other species usually receive little or no attention, which points to the need for more extensive research on this issue.

The origin of PTEs in the studied mushrooms is questionable. Results of the BCF assessment indicated that all the species are strong accumulators of Cd, Cu, and Zn from the soil (Table S5 and S6). However, the general absence of stronger relationships between the soil and mushroom PTE contents (moderate correlation in the whole data set was found only for Pb; Table S7) suggests the possibility of another source. Although BCF values > 1 are common for mushrooms, which can reach values in the order of tens^18^ to hundreds^11^, extreme values as in Cd (BCF > 1000) indicate the importance of another accumulation mechanism than the uptake from bulk soil.

The life cycle of mushrooms is more dynamic and more easily influenced by short-term (current) environmental conditions compared to soil. Thus, it can be assumed that wet atmospheric deposition of PTEs associated with both vertical and horizontal precipitation, may also have an impact. Mushrooms' accumulation potential^12,26^ enables them to rapidly and efficiently take up and store soil solution-bound atmospheric depositions, thus accelerating the Cd cycle in particular. Consequently, Cd does not accumulate in the soil, as Cd contents were below the detection limit in 59% of the soil samples. On the other hand, in mushrooms, Cd was found in all samples, often at levels typical for areas with serious soil pollution^10,13^. The soil contents of Cd in the studied area do not reach the values of contaminated areas even in the potentially most contaminated forest floor^30^ from which mushrooms also take up nutrients. Without a direct site-related source of Cd, only precipitation-related atmospheric deposition can be a predominant source. Although direct atmospheric deposition is typically considered to be of minor importance due to the short lifespan of fruiting bodies in most edible mushroom species^46^, the results of this study may question this assumption as they suggest the opposite.

Due to species-dependent variation in PTEs accumulation^17,46,47^ (Table 2 and S4), *B. edulis*, *X. chrysenteron,* and *I. badia* were tested to verify the effect of beech and spruce on the accumulation of PTEs in mushrooms. The results mostly did not confirm any significant importance of the forest type for these mushroom species contents of PTEs (Table 3). However, analysis of the whole dataset of mushrooms or separately for the two forest types revealed stronger correlations for particular forest types (Table S7), especially in the case of Pb (*r* = 0.4138 =  > EB *r* = 0.5408 and NS *r* = 0.4390) and Zn (*r* = 0.0226 =  > EB *r* = −0.3792 and NS *r* = 0.4223). For Zn, this differentiation led to a reverse relationship in beech as compared to the spruce stands, suggesting differences in the biogeochemical cycling of this element depending on the tree species. These findings point to the need for further research on this issue.

**Table 3:** Average PTE contents (mg/kg) of the most abundant mushroom species (> 5 samples for each forest type); different letters indicate significant differences in the PTE content of the species between forest types (Mann–Whitney U Test, *p* = 0.05).

Mushroom species	Forest type	Cd	Cu	Pb	Zn
*Imleria badia*	Beech	3.56	25.7 a	1.40 a	215 a
	Spruce	2.26	30.9 a	0.37 b	222 a
*Xerocomellus chrysenteron*	Beech	5.47	15.3 b	2.88 a	124 a
	Spruce	5.79	24.4 a	3.41 a	174 a
*Boletus edulis*	Beech	7.53	33.4 a	1.28 a	264 a
	Spruce	4.69	32.1 a	0.92 a	284 a

### Health risk assessment of mushrooms

**Figure 3:**
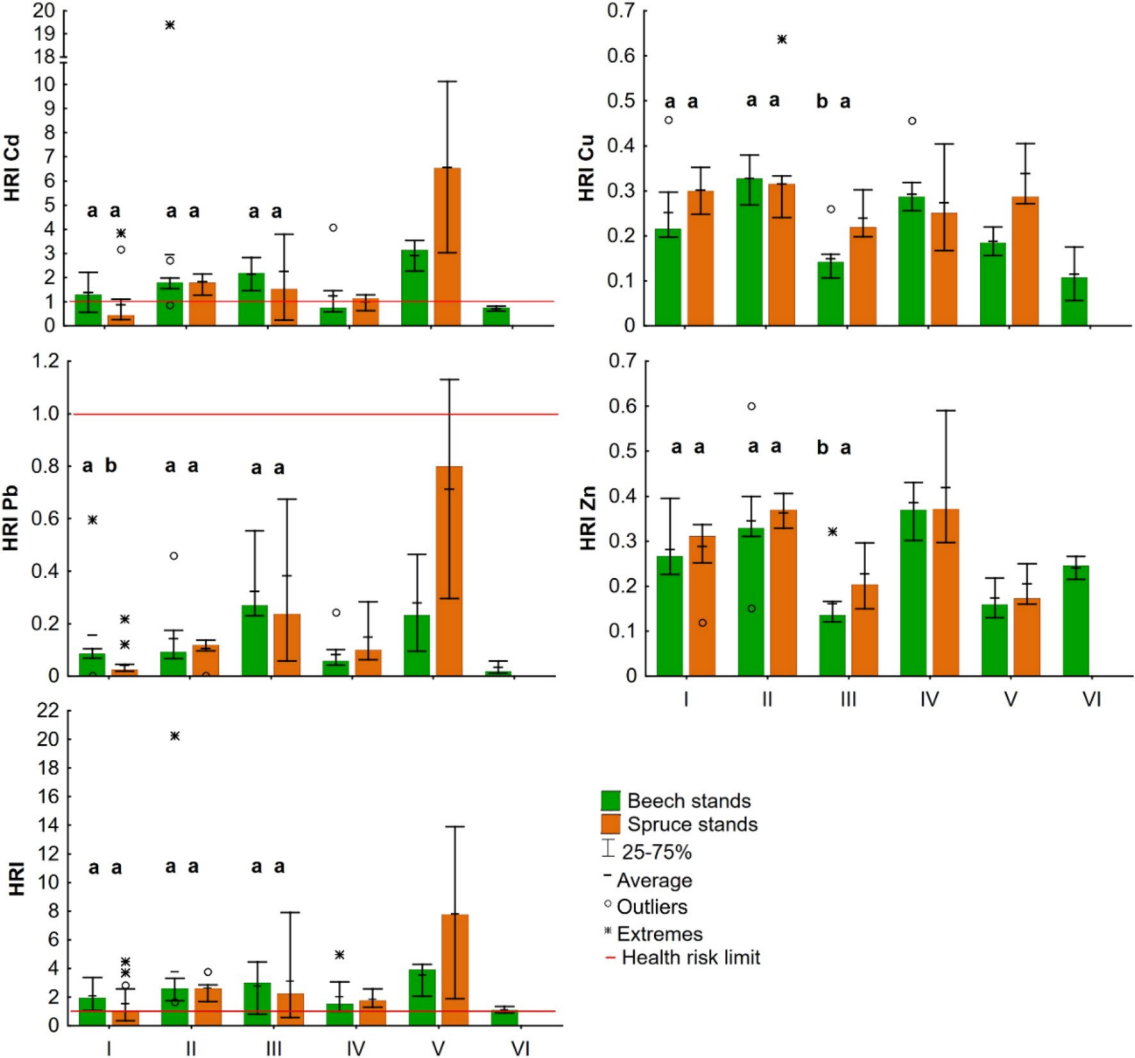
Median HRIs of the PTEs and the total HRI for (I) *Imleria badia*, (II) *Boletus edulis*, (III) *Xerocomellus chrysenteron*, (IV) *Neoboletus luridiformis*, (V) *Xerocomellus pruinatus*, and (VI) *Xerocomus subtomentosus*; different lowercase letters indicate significant differences between forest types in HRI of mushroom species with enough samples for statistical analysis (Mann–Whitney U test, *p* = 0.05).

The total HRI for all PTEs (defined only for easier summarization and orientation) exceeded the limit value, indicating a potential health risk in 82% of mushroom samples (Table S5). Due to its toxicity^31,36,39^, Cd had the largest contribution to this risk, exceeding the limit value in 59% of the cases (Fig. 3). Spatial distribution of the mushrooms-related health risk assessment is presented in Figure S1A–D.

The results showed *X. pruinatus* as potentially the riskiest species for consumption, in which the average HRI value reached 5.69, mainly due to the high levels of Cd (HRI_Cd_ = 4.74). However, due to the limited number of samples and the absence of studies on contamination and health risk of this species, further research is needed to verify this finding. The significance of Cd-related risk is also evidenced by the fact that such a high HRI was not achieved for any PTE in the 19 mushroom species studied by Sarikurkcu et al.^38^. However, some species of the *Boletaceae* family can reach even higher HRI_Cd_ values^12^.

*Boletaceae edulis* and *X. chrysenterone,* with HRI values of 3.33 and 3.01, respectively, also pose a high risk. As these two species are among the most abundant from their family in Central European forests, the risk of poisoning is worthy of interest. Furthermore, *B. edulis* belongs among the widely consumed species^18^. *N. luridiformis* (HRI = 1.98), *I. badia* (HRI = 1.66), and *X. subtomentosus* (HRI = 1.11) pose a lower risk. Similarly, Komárek et al.^10^ report *B. edulis*, *I. badia,* and *X. chrysenteron* as the species whose consumption may pose a significant toxicological risk. Their study, however, was focused on the area contaminated by the lead smelter.

The influence of beech and spruce on the potential health risk of mushrooms was significant (Fig. 3) only in the case of *I. badia* (HRI_Pb_) and *X. chrysenteron* (HRI_Cu_, HRI_Zn_). The visible potential influence of forest type on the other species cannot be statistically verified due to the small sample size. In general, no significant differences were found between the forest types for any of the PTEs (Mann–Whitney U Test, *p* > 0.05). The mushroom species and related ability to accumulate a given element (Fig. 3) appear to be a more important factor in the overall health risk assessment. Nevertheless, based on the results (Table 3 and S7; Fig. 3), the effect of forest type on PTEs levels in mushrooms and their health risks can be considered significant. Hypothesis H3, however, cannot be confirmed.

## Conclusions

A soil pollution assessment classified the studied forest soils as safe. Although there were no significant differences between the forest types in the average topsoil contents of PTEs, the correlations of PTEs contents to soil C contents showed different results for particular forest types. Unlike the spruce-dominated stands, the PTEs contents in beech stands showed significant positive correlations with soil C contents. Despite the absence of soil pollution, serious contamination was found in mushrooms as Cd and Zn exceeded the Czech hygienic limits in 66 and 100% of the samples, respectively, and the total PTEs content represented a potential health risk in 82% of the samples. Thus, even mushrooms in forests without a local source of contamination and soil pollution can be hazardous. Other sources, such as direct and indirect uptake of PTEs from vertical and horizontal precipitation and throughfall (in addition to soil), should be considered. The results on the forest type influence on the accumulation of PTEs in common mushrooms are inconclusive; however, in the general evaluation, they confirm the significant influence of the tree species, especially in the case of Zn. These findings point to the need for further research on this issue. The results suggest that for spatial modelling of PTEs pollution in forest soils, not only the interpolation of data based on random sampling in forest stands is sufficient, but it is also necessary to consider the factor of tree species and its representation in forest stands due to their significant influence on other soil properties essential for the behaviour of PTEs.

## Supplementary Information

Supplementary Information.
