# Supplementary material for: Influence of beech and spruce on potentially toxic elements-related health risk of edible mushrooms growing on unpolluted forest soils
Source: Sci Rep. 2022 Mar 30;12:5407. doi: 10.1038/s41598-022-09400-9 (PMC8967844; doi:10.1038/s41598-022-09400-9)
Supplement: Supplementary file 1 — Supplementary Information. [file 41598_2022_9400_MOESM1_ESM.docx]

**Supplementary material**

**Influence of beech and spruce on potentially toxic elements-related health risk of edible mushrooms growing on unpolluted forest soils**

Václav Pecina ^1, 2^, Martin Valtera ^3^, Karel Drápela ^4^, Radek Novotný ^5^, Petr Vahalík ^4^, Renata Komendová ^1^, Martin Brtnický ^1, 2^, David Juřička ^3 *^

*^1^ Institute of Chemistry and Technology of Environmental Protection, Faculty of Chemistry, Brno University of Technology, Purkyňova 118, 61200 Brno, Czech Republic*

*^2^ Department of Agrochemistry, Soil Science, Microbiology and Plant Nutrition, Faculty of AgriSciences, Mendel University in Brno, Zemědělská 1, 613 00 Brno, Czech Republic*

*^3^ Department of Geology and Soil Science, Faculty of Forestry and Wood Technology, Mendel University in Brno, Zemědělská 3, 613 00 Brno, Czech Republic*

*^4^ Department of Forest Management and Applied Geoinformatics, Faculty of Forestry and Wood Technology, Mendel University in Brno, Zemědělská 3, 613 00 Brno, Czech Republic*

*^5^ Forestry and Game Management Research Institute, Strnady 136, 252 02 Jíloviště, Czech Republic*

** Corresponding Author,* e-mail [david.juricka@mendelu.cz](mailto:david.juricka@mendelu.cz); ORCID 0000-0002-7895-589X

**Table S1**. The list of studied mushroom species

| **Species** | **Number of samples** | | |
| --- | --- | --- | --- |
|  | **Beech** | **Spruce** | **Total** |
| *Imleria badia* (Fr.) Vizzini | 6 | 21 | 27 |
| *Boletus edulis* Bull. | 14 | 9 | 23 |
| *Xerocomellus chrysenteron* (Bull.) Šutara | 7 | 14 | 21 |
| *Neoboletus luridiformis* (Rostk.) Gelardi, Simonini & Vizzini | 8 | 3 | 11 |
| *Xerocomellus pruinatus* (Fr. & Hök) Šutara | 4 | 4 | 8 |
| *Xerocomus subtomentosus* (L.) Quél. | 4 | 0 | 4 |
| *Cyanoboletus pulverulentus* (Opat.) Gelardi, Vizzini & Simonini | 1 | 0 | 1 |
| *Porphyrellus porphyrosporus* (Fr. & Hök) E.-J. Gilbert | 1 | 0 | 1 |

**Table S2**. Detailed information on the quality assurance and quality control of the analysis of potentially toxic elements (PTEs)

|  | **Range of relative standard deviations of the triplicate measurements (%)** | | **Range of recoveries (%)** | | |
| --- | --- | --- | --- | --- | --- |
|  | **Soil** | **Mushrooms** | **METRANAL 31** | **METRANAL 33** | **METRANAL 34** |
| **Cd** | 0.9–6.8 | 0.5–5.9 | 93.5–112.3 | 94.2–97.4 | 96.3–102.7 |
| **Cu** | 0.6–4.9 | 0.1–5.6 | 98.6–101.7 | 97.7–103.2 | 99.5–100.4 |
| **Pb** | 0.5–6.3 | 0.2–8.3 | 96.7–106.6 | 95.6–103.6 | 97.5–102.4 |
| **Zn** | 0.8–5.7 | 0.4–3.2 | 97.2–102.9 | 97.7–102.6 | 98.5–102.9 |

**Table S3**. Soil average (±S.D.) PTEs contents (mg/kg) and pollution assessment by the Integrated Nemerow Pollution Index (IPI_N_)

|  | **PTE** | | | | **IPI_N_** | |
| --- | --- | --- | --- | --- | --- | --- |
|  | **Cd** | **Cu** | **Pb** | **Zn** | **Dutch standard^1^** | **Czech standard^2^** |
| Overall | 0.024±0.032 | 12.8±5.94 | 13.6±15.2 | 84.9±34.7 | 0.48 | 0.56 |
| Beech | 0.027±0.029 | 13.5±6.42 | 14.4±18.7 | 92.1±33.9 | 0.52 | 0.59 |
| Spruce | 0.021±0.033 | 12.1±5.35 | 13.0±10.8 | 78.0±34.0 | 0.45 | 0.52 |
| Dutch limit^I^ | 0.80 | 36.0 | 85.0 | 140 | - | - |
| Czech limit^II^ | 0.50 | 60.0 | 60.0 | 120 | - | - |

^I^ VROM^1^

^II^ Decree No. 153/2016 Coll.^2^

**Table S4**. Coordinates of sampling sites and soil PTEs contents there

| **Site No.** | **Tree species** | **Coordinates (°)** | | **PTE content (mg/kg)** | | | |
| --- | --- | --- | --- | --- | --- | --- | --- |
|  |  | **Latitude** | **Longitude** | **Cd** | **Cu** | **Pb** | **Zn** |
| 1 | Beech | 50.37273 | 16.99597 | 0.028 | 10.1 | 2.82 | 92.3 |
| 2 |  | 50.33433 | 16.94496 | 0.008 | 4.27 | 1.61 | 51.1 |
| 3 |  | 50.33265 | 16.94880 | 0.014 | 7.05 | 2.87 | 68.2 |
| 4 |  | 50.25834 | 17.41442 | 0.081 | 15.9 | 9.94 | 104 |
| 5 |  | 50.19058 | 17.13671 | 0.058 | 14.9 | 8.35 | 80.6 |
| 6 |  | 50.23845 | 17.38980 | 0.085 | 24.7 | 10.7 | 133 |
| 7 |  | 50.19866 | 17.12979 | 0.070 | 9.36 | 6.27 | 60.5 |
| 8 |  | 50.32079 | 17.00700 | 0.027 | 3.84 | 1.06 | 28.2 |
| 9 |  | 50.31091 | 17.00496 | <0.003 | 4.95 | 1.92 | 66.4 |
| 10 |  | 50.13812 | 17.21416 | 0.027 | 16.3 | 5.66 | 107 |
| 11 |  | 50.14575 | 17.22432 | 0.021 | 16.2 | 4.28 | 81.9 |
| 12 |  | 50.17577 | 17.23609 | 0.016 | 14.5 | 9.85 | 113 |
| 13 |  | 50.27525 | 17.04413 | 0.050 | 15.9 | 1.89 | 102 |
| 14 |  | 50.16029 | 17.15540 | <0.003 | 12.7 | 3.00 | 46.2 |
| 15 |  | 50.29523 | 17.21573 | 0.087 | 14.3 | 3.55 | 84.7 |
| 16 |  | 50.25676 | 17.06673 | 0.060 | 18.8 | 7.81 | 111 |
| 17 |  | 50.27996 | 17.17805 | 0.021 | 7.68 | 1.86 | 63.9 |
| 18 |  | 50.27631 | 17.14919 | <0.003 | 6.87 | 2.64 | 49.9 |
| 19 |  | 50.28595 | 17.20542 | 0.002 | 20.8 | 5.80 | 111 |
| 20 |  | 50.12888 | 17.22761 | <0.003 | 28.2 | 40.2 | 128 |
| 21 |  | 50.22786 | 17.06562 | <0.003 | 12.6 | 51.2 | 106 |
| 22 |  | 50.20413 | 17.10963 | <0.003 | 11.2 | 66.6 | 119 |
| 23 |  | 50.34729 | 16.96453 | <0.003 | 9.22 | 16.0 | 80.7 |
| 24 |  | 50.26020 | 17.04053 | <0.003 | 11.7 | 47.5 | 126 |
| 25 |  | 50.25179 | 17.09187 | <0.003 | 25.8 | 45.8 | 187 |
| 26 | Spruce | 50.35682 | 16.96896 | <0.003 | 12.0 | 8.99 | 135 |
| 27 |  | 50.34362 | 16.94388 | <0.003 | 8.52 | 5.39 | 90.9 |
| 28 |  | 50.23904 | 17.26473 | <0.003 | 8.53 | 4.53 | 50.3 |
| 29 |  | 50.32984 | 16.94058 | <0.003 | 6.44 | 6.94 | 49.2 |
| 30 |  | 50.31196 | 16.99076 | <0.003 | 10.5 | 13.7 | 91.1 |
| 31 |  | 50.24323 | 17.39163 | 0.062 | 10.7 | 6.73 | 86.3 |
| 32 |  | 50.25636 | 17.02381 | 0.090 | 17.4 | 8.62 | 106 |
| 33 |  | 50.20383 | 17.14143 | 0.021 | 14.4 | 3.01 | 63.0 |
| 34 |  | 50.20956 | 17.33131 | 0.075 | 11.1 | 11.4 | 44.1 |
| 35 |  | 50.26870 | 17.04145 | 0.021 | 15.6 | 5.54 | 102 |
| 36 |  | 50.25211 | 17.09447 | <0.003 | 19.6 | 18.2 | 137 |
| 37 |  | 50.33008 | 16.96395 | <0.003 | 4.26 | 5.03 | 26.2 |
| 38 |  | 50.14707 | 17.21120 | 0.123 | 7.93 | 8.34 | 45.4 |
| 39 |  | 50.27427 | 17.13245 | <0.003 | 8.40 | 8.18 | 86.2 |
| 40 |  | 50.26054 | 17.13879 | <0.003 | 13.2 | 2.65 | 115 |
| 41 |  | 50.21310 | 17.30286 | 0.093 | 11.2 | 8.93 | 79.3 |
| 42 |  | 50.18679 | 17.23289 | <0.003 | 12.6 | 7.76 | 119 |
| 43 |  | 50.23187 | 17.22604 | <0.003 | 9.29 | 11.6 | 55.7 |
| 44 |  | 50.24904 | 17.38251 | 0.012 | 28.3 | 6.65 | 142 |
| 45 |  | 50.12329 | 17.18337 | 0.052 | 11.9 | 22.0 | 61.3 |
| 46 |  | 50.14215 | 17.18121 | <0.003 | 9.38 | 20.2 | 38.1 |
| 47 |  | 50.13288 | 17.22379 | <0.003 | 23.6 | 32.6 | 93.4 |
| 48 |  | 50.22284 | 17.05865 | 0.053 | 10.9 | 31.4 | 80.6 |
| 49 |  | 50.30361 | 17.01595 | 0.021 | 6.81 | 49.7 | 29.3 |
| 50 |  | 50.18855 | 17.15312 | <0.003 | 15.0 | 21.4 | 27.5 |
| 51 |  | 50.35822 | 16.96946 | <0.003 | 6.12 | 7.50 | 74.5 |

**Table S5.** Summary of PTEs contents (mg/kg) in mushrooms, mushrooms bioconcentration factors (BCF), and their total potential health risks (HRI)

| **Forest type** | **Mushroom species** | **PTE content** | | | | **BCF** | | | | **HRI** |
| --- | --- | --- | --- | --- | --- | --- | --- | --- | --- | --- |
|  |  | **Cd** | **Cu** | **Pb** | **Zn** | **Cd** | **Cu** | **Pb** | **Zn** |  |
| Beech | *Boletus edulis* | 3.92 | 36.1 | 0.86 | 219 | 509 | 8.45 | 0.54 | 4.30 | 2.28 |
|  |  | 5.04 | 27.4 | 1.55 | 253 | 61.9 | 1.72 | 0.16 | 2.43 | 2.75 |
|  |  | 2.65 | 33.3 | 0.61 | 238 | 31.1 | 1.35 | 0.06 | 1.79 | 1.75 |
|  |  | 5.02 | 30.3 | 0.73 | 309 | 186 | 7.91 | 0.69 | 11.0 | 2.76 |
|  |  | 2.18 | 33.4 | 0.41 | 320 | 81.8 | 2.05 | 0.07 | 2.99 | 1.65 |
|  |  | 4.06 | 38.7 | 1.49 | 237 | 261 | 2.68 | 0.15 | 2.10 | 2.45 |
|  |  | 5.26 | 17.1 | 0.44 | 202 | 105 | 1.07 | 0.23 | 1.98 | 2.55 |
|  |  | 6.92 | 22.8 | 0.50 | 249 | 138 | 1.43 | 0.26 | 2.45 | 3.32 |
|  |  | 2.81 | 47.2 | 2.56 | 305 | 32.2 | 3.31 | 0.72 | 3.60 | 2.25 |
|  |  | 4.83 | 36.5 | 1.73 | 288 | 81.1 | 1.95 | 0.22 | 2.59 | 2.83 |
|  |  | 4.82 | 39.4 | 0.60 | 245 | 225 | 5.12 | 0.32 | 3.84 | 2.67 |
|  |  | 4.33 | 30.1 | 1.50 | 253 | 2,889 | 4.37 | 0.57 | 5.07 | 2.50 |
|  |  | 4.25 | 49.2 | 0.77 | 458 | 2,361 | 2.37 | 0.13 | 4.14 | 2.84 |
|  |  | 49.4 | 26.2 | 4.09 | 115 | 32,933 | 0.93 | 0.10 | 0.90 | 20.3 |
| Spruce |  | 5.46 | 18.9 | 1.05 | 245 | 3,642 | 1.58 | 0.12 | 1.81 | 2.77 |
|  |  | 3.23 | 65.0 | 0.77 | 319 | 2,151 | 7.62 | 0.17 | 6.34 | 2.41 |
|  |  | 2.61 | 33.9 | 0.86 | 258 | 1,743 | 3.23 | 0.06 | 2.83 | 1.79 |
|  |  | 2.50 | 24.9 | 1.26 | 251 | 40.3 | 2.33 | 0.19 | 2.91 | 1.70 |
|  |  | 3.50 | 18.3 | 1.11 | 310 | 2,335 | 4.29 | 0.22 | 11.8 | 2.08 |
|  |  | 4.68 | 24.5 | 1.32 | 282 | 3,122 | 2.91 | 0.16 | 3.27 | 2.60 |
|  |  | 5.08 | 33.1 | 1.22 | 306 | 3,384 | 2.64 | 0.16 | 2.56 | 2.86 |
|  |  | 7.22 | 39.4 | 0.86 | 343 | 583 | 1.39 | 0.13 | 2.42 | 3.77 |
|  |  | 8.17 | 32.1 | 0.02 | 186 | 157 | 2.71 | 0.00 | 3.04 | 3.77 |
| Beech | *Cyanoboletus pulverulentus* | 0.34 | 12.0 | 0.11 | 179 | 12.4 | 1.18 | 0.04 | 1.93 | 0.50 |
| Beech | *Imleria badia* | 3.14 | 20.1 | 0.80 | 173 | 408 | 4.71 | 0.50 | 3.38 | 1.75 |
|  |  | 5.63 | 22.3 | 0.73 | 307 | 397 | 3.15 | 0.25 | 4.50 | 2.91 |
|  |  | 1.32 | 30.2 | 0.61 | 302 | 734 | 1.45 | 0.11 | 2.73 | 1.28 |
|  |  | 1.44 | 21.5 | 0.02 | 217 | 957 | 1.71 | 0.00 | 2.04 | 1.06 |
|  |  | 3.40 | 46.7 | 0.93 | 190 | 2,265 | 4.18 | 0.01 | 1.59 | 2.14 |
|  |  | 6.41 | 13.4 | 5.32 | 99.7 | 4,270 | 1.14 | 0.11 | 0.79 | 3.37 |
| Spruce |  | 0.63 | 49.1 | 0.23 | 276 | 421 | 4.10 | 0.03 | 2.04 | 1.12 |
|  |  | 1.81 | 35.9 | 0.24 | 266 | 1,209 | 4.21 | 0.05 | 2.92 | 1.44 |
|  |  | 0.23 | 31.0 | 0.13 | 212 | 156 | 3.63 | 0.03 | 4.21 | 0.69 |
|  |  | 1.25 | 30.1 | 0.58 | 247 | 835 | 2.86 | 0.04 | 2.72 | 1.18 |
|  |  | 1.09 | 30.5 | 0.13 | 229 | 17.6 | 2.86 | 0.02 | 2.66 | 1.04 |
|  |  | 0.77 | 41.1 | 0.14 | 270 | 854 | 2.36 | 0.02 | 2.54 | 1.07 |
|  |  | 1.20 | 14.5 | 0.48 | 153 | 571 | 1.01 | 0.16 | 2.42 | 0.87 |
|  |  | 2.18 | 29.0 | 0.22 | 246 | 29.0 | 2.61 | 0.02 | 5.58 | 1.49 |
|  |  | 0.76 | 28.9 | 0.20 | 257 | 35.8 | 1.86 | 0.04 | 2.53 | 0.94 |
|  |  | 0.23 | 35.7 | 0.27 | 291 | 151 | 1.82 | 0.01 | 2.13 | 0.85 |
|  |  | 2.82 | 33.2 | 0.40 | 238 | 1,881 | 7.80 | 0.08 | 9.06 | 1.79 |
|  |  | 4.70 | 37.6 | 0.18 | 257 | 38.3 | 4.74 | 0.02 | 5.67 | 2.57 |
|  |  | 0.60 | 19.6 | 0.23 | 244 | 400 | 2.33 | 0.03 | 2.83 | 0.77 |
|  |  | 0.60 | 44.6 | 0.20 | 277 | 400 | 3.39 | 0.07 | 2.40 | 1.06 |
|  |  | 5.51 | 33.8 | 0.39 | 226 | 59.1 | 3.03 | 0.04 | 2.85 | 2.84 |
|  |  | 8.07 | 28.9 | 0.11 | 185 | 155 | 2.43 | 0.00 | 3.01 | 3.70 |
|  |  | 9.78 | 38.0 | 0.19 | 192 | 6,521 | 4.05 | 0.01 | 5.05 | 4.49 |
|  |  | 0.17 | 15.0 | 0.02 | 91.1 | 117 | 0.64 | 0.00 | 0.98 | 0.34 |
|  |  | 1.10 | 21.2 | 1.94 | 144 | 20.8 | 1.94 | 0.06 | 1.78 | 1.05 |
|  |  | 4.13 | 23.7 | 0.54 | 142 | 200 | 3.48 | 0.01 | 4.82 | 2.10 |
|  |  | 0.90 | 25.3 | 1.08 | 196 | 597 | 1.69 | 0.05 | 7.15 | 0.98 |
| Beech | *Neoboletus luridiformis* | 2.69 | 17.2 | 0.41 | 211 | 349 | 4.03 | 0.26 | 4.13 | 1.55 |
|  |  | 1.87 | 31.5 | 0.60 | 300 | 32.5 | 2.11 | 0.07 | 3.72 | 1.50 |
|  |  | 10.4 | 27.0 | 2.17 | 291 | 6,920 | 5.45 | 1.13 | 4.38 | 4.96 |
|  |  | 1.92 | 33.3 | 0.72 | 274 | 71.8 | 2.04 | 0.13 | 2.57 | 1.52 |
|  |  | 1.69 | 31.6 | 0.33 | 358 | 108 | 2.18 | 0.03 | 3.16 | 1.48 |
|  |  | 4.75 | 46.5 | 1.08 | 465 | 3,165 | 3.66 | 0.36 | 10.1 | 3.05 |
|  |  | 1.24 | 26.3 | 0.19 | 249 | 14.2 | 1.85 | 0.05 | 2.94 | 1.09 |
|  |  | 1.08 | 26.0 | 0.44 | 213 | 602 | 1.25 | 0.08 | 1.92 | 1.01 |
| Spruce |  | 3.26 | 41.1 | 2.53 | 451 | 2,171 | 4.83 | 0.47 | 4.96 | 2.56 |
|  |  | 2.91 | 17.0 | 0.56 | 284 | 1,939 | 2.64 | 0.08 | 5.77 | 1.74 |
|  |  | 1.61 | 25.6 | 0.89 | 227 | 1,075 | 2.75 | 0.08 | 4.07 | 1.28 |
| Spruce | *Porphyrellus porphyrosporus* | 1.12 | 8.9 | 0.34 | 307 | 744 | 1.06 | 0.04 | 3.56 | 0.97 |
| Beech | *Xerocomellus chrysenteron* | 0.50 | 26.5 | 0.11 | 246 | 18.1 | 2.61 | 0.04 | 2.66 | 0.79 |
|  |  | 5.56 | 15.4 | 4.94 | 92.1 | 3,704 | 0.55 | 0.12 | 0.72 | 3.01 |
|  |  | 5.15 | 16.2 | 2.39 | 103 | 3,432 | 1.29 | 0.05 | 0.97 | 2.58 |
|  |  | 3.72 | 10.9 | 2.15 | 127 | 2,481 | 0.97 | 0.03 | 1.07 | 1.98 |
|  |  | 7.13 | 10.6 | 3.18 | 85.0 | 4,751 | 1.15 | 0.20 | 1.05 | 3.37 |
|  |  | 9.08 | 14.4 | 5.33 | 113 | 6,056 | 1.23 | 0.11 | 0.89 | 4.45 |
|  |  | 7.18 | 13.3 | 2.06 | 100 | 4,786 | 0.51 | 0.04 | 0.54 | 3.31 |
| Spruce |  | 2.34 | 26.6 | 0.72 | 263 | 1,562 | 2.22 | 0.08 | 1.94 | 1.61 |
|  |  | 0.46 | 20.1 | 0.93 | 182 | 306 | 2.36 | 0.17 | 2.00 | 0.72 |
|  |  | 0.59 | 33.2 | 0.63 | 226 | 28.0 | 2.13 | 0.11 | 2.23 | 0.93 |
|  |  | 0.05 | 24.6 | 0.35 | 281 | 34.9 | 1.26 | 0.02 | 2.06 | 0.67 |
|  |  | 0.23 | 15.9 | 0.52 | 205 | 155 | 1.89 | 0.06 | 2.37 | 0.57 |
|  |  | 1.57 | 30.8 | 0.40 | 288 | 1,044 | 2.34 | 0.15 | 2.50 | 1.34 |
|  |  | 0.76 | 20.2 | 0.38 | 180 | 509 | 1.61 | 0.05 | 1.51 | 0.78 |
|  |  | 13.3 | 32.3 | 8.75 | 115 | 255 | 2.72 | 0.40 | 1.87 | 6.67 |
|  |  | 15.8 | 32.4 | 10.8 | 126 | 10,540 | 3.45 | 0.53 | 3.32 | 7.90 |
|  |  | 5.40 | 22.8 | 3.29 | 130 | 3,599 | 0.97 | 0.10 | 1.39 | 2.88 |
|  |  | 6.73 | 20.2 | 3.55 | 106 | 127 | 1.85 | 0.11 | 1.31 | 3.38 |
|  |  | 9.66 | 21.8 | 4.63 | 99.3 | 183 | 2.00 | 0.15 | 1.23 | 4.66 |
|  |  | 15.5 | 21.0 | 6.01 | 131 | 754 | 3.08 | 0.12 | 4.47 | 7.15 |
|  |  | 8.62 | 20.3 | 6.80 | 107 | 5,746 | 1.36 | 0.32 | 3.88 | 4.49 |
| Beech | *Xerocomellus pruinatus* | 9.47 | 21.5 | 0.89 | 190 | 457 | 1.33 | 0.21 | 2.33 | 4.28 |
|  |  | 4.18 | 15.8 | 0.80 | 143 | 268 | 1.09 | 0.08 | 1.27 | 2.07 |
|  |  | 8.56 | 23.3 | 5.04 | 97.5 | 5,709 | 2.52 | 0.32 | 1.21 | 4.28 |
|  |  | 7.41 | 16.0 | 3.24 | 100 | 4,939 | 0.62 | 0.07 | 0.54 | 3.56 |
| Spruce |  | 3.21 | 28.3 | 0.12 | 245 | 2,140 | 2.15 | 0.05 | 2.12 | 1.87 |
|  |  | 30.5 | 52.3 | 11.0 | 137 | 585 | 4.40 | 0.50 | 2.23 | 13.9 |
|  |  | 12.2 | 27.1 | 9.10 | 117 | 8,147 | 2.89 | 0.45 | 3.07 | 6.24 |
|  |  | 21.0 | 30.3 | 5.14 | 127 | 1,020 | 4.45 | 0.10 | 4.34 | 9.29 |
| Beech | *Xerocomus subtomentosus* | 1.38 | 7.2 | 0.06 | 202 | 19.7 | 0.77 | 0.01 | 3.34 | 0.88 |
|  |  | 2.05 | 21.0 | 0.12 | 157 | 99.0 | 1.30 | 0.03 | 1.91 | 1.23 |
|  |  | 1.73 | 4.3 | 0.17 | 173 | 34.4 | 0.27 | 0.09 | 1.70 | 0.97 |
|  |  | 2.14 | 14.6 | 0.85 | 205 | 1,427 | 2.13 | 0.32 | 4.10 | 1.35 |

**Table S6**. Bioconcentration factors (BCF) of the mushrooms

| **Species** | **Cd** | | **Cu** | | **Pb** | | **Zn** | |
| --- | --- | --- | --- | --- | --- | --- | --- | --- |
|  | **Average** | **S.D.** | **Average** | **S.D.** | **Average** | **S.D.** | **Average** | **S.D.** |
| *Imleria badia* (n = 28) | 878 | 1,422 | 2.93 | 1.48 | 0.07 | 0.10 | 3.35 | 1.8 |
| *Boletus edulis* (n = 24) | 2,480 | 6,612 | 3.19 | 2.10 | 0.24 | 0.20 | 3.75 | 2.6 |
| *Xerocomellus chrysenteron* (n = 21) | 2,384 | 2,726 | 1.79 | 0.79 | 0.14 | 0.13 | 1.90 | 1.0 |
| *Neoboletus luridiformis* (n = 11) | 1,495 | 1,987 | 2.98 | 1.27 | 0.25 | 0.31 | 4.33 | 2.1 |
| *Xerocomellus pruinatus* (n = 8) | 2,908 | 2,783 | 2.43 | 1.30 | 0.22 | 0.17 | 2.14 | 1.1 |
| *Xerocomus subtomentosus* (n = 4) | 395 | 596 | 1.12 | 0.69 | 0.11 | 0.12 | 2.76 | 1.0 |

**Table S7**. Linear regression models of pairs of PTEs contents in soil (independent variable) and mushrooms (dependent variable); separate statistics are provided for the whole dataset and for particular forest types

| Variable | Whole dataset | | | Spruce stands | | | Beech stands | | |
| --- | --- | --- | --- | --- | --- | --- | --- | --- | --- |
|  | *p*-value | *r* | *r^2^* | *p*-value | *r* | *r^2^* | *p*-value | *r* | *r^2^* |
| Cu | 0.8664 | -0.0176 | 0.0003 | 0.8795 | 0.0218 | 0.0005 | 0.9084 | 0.0181 | 0.0003 |
| Pb | **0.0000** | 0.4138 | 0.1712 | **0.0013** | 0.4390 | 0.1927 | **0.0002** | 0.5408 | 0.2925 |
| Zn | 0.8285 | 0.0226 | 0.0005 | **0.0020** | 0.4223 | 0.1783 | **0.0122** | -0.3792 | 0.1438 |


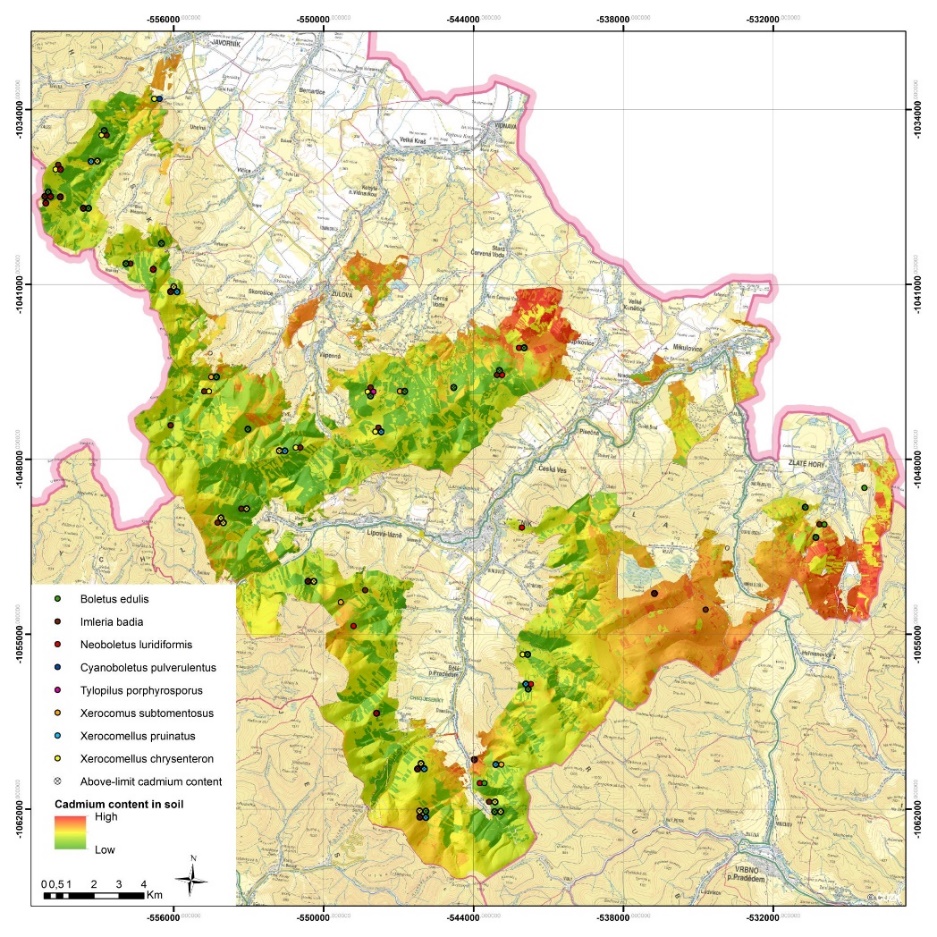

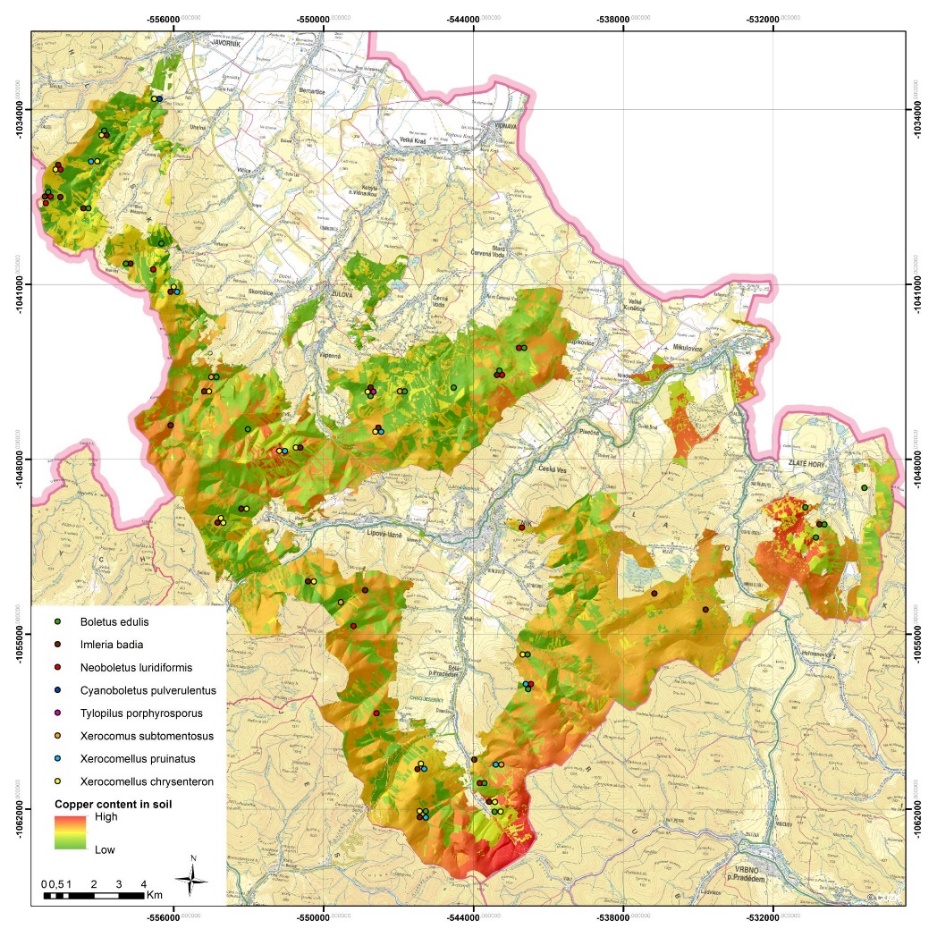


**(B)**

**(A)**

**Figure S1**. Spatial distribution of PTEs in soil, including identification of the occurrence of potentially hazardous mushrooms for Cd (A), Cu (B), Pb (C), and Zn (D). The range of PTE values in soil presented in individual maps is: Cd 0.03–0.13 mg/kg, Cu 0.97–48.5 mg/kg, Pb 1.06–81.5 mg/kg, and Zn 12.2–240 mg/kg


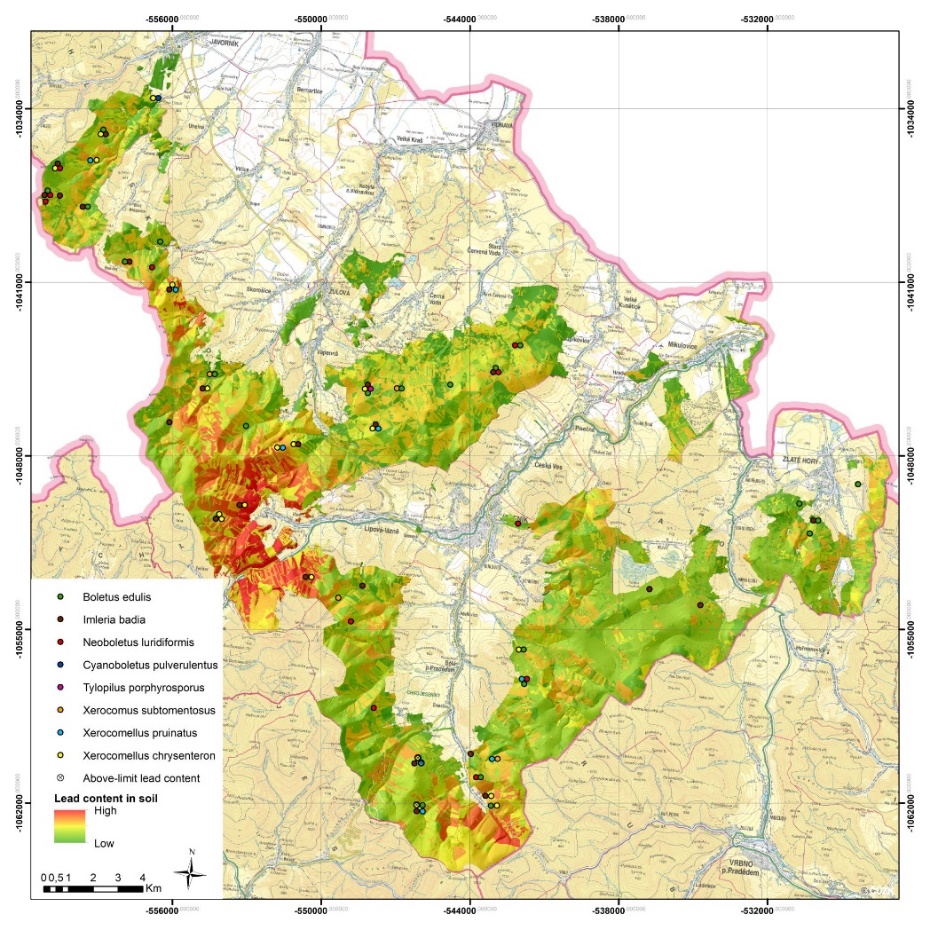

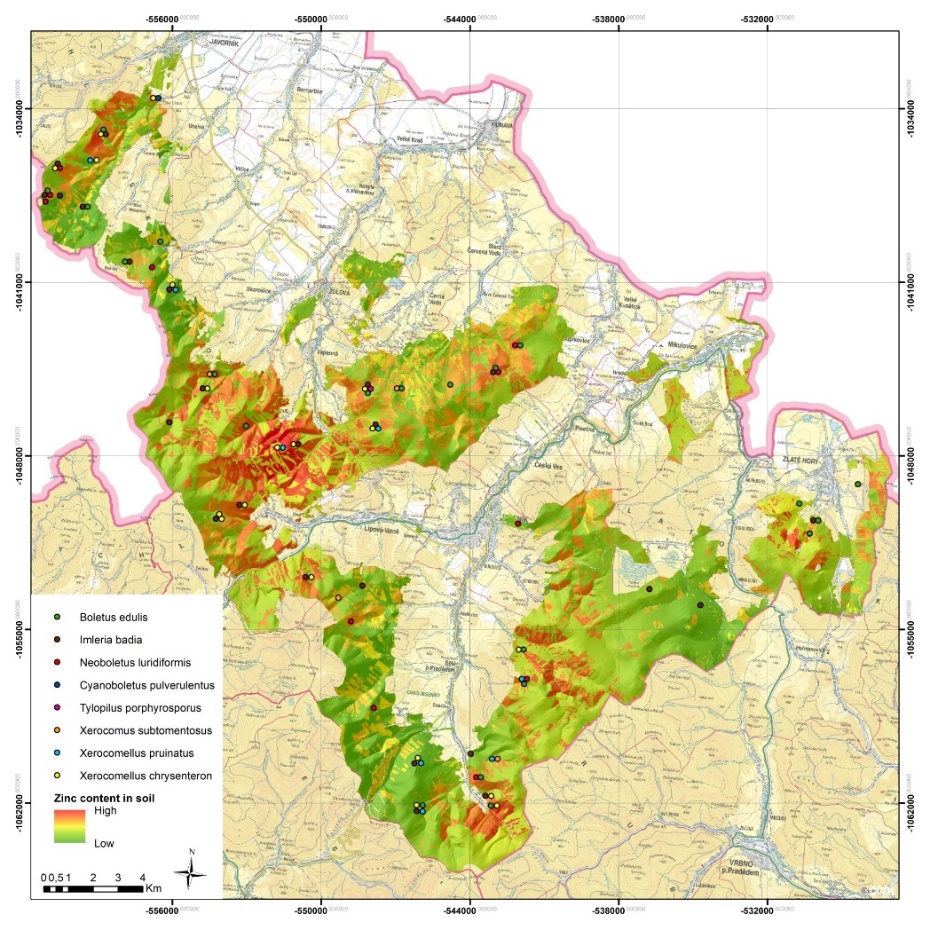


**(D)**

**(C)**

**References**

1. VROM. *Circular on Target Values and Intervention Values for Soil Remediation*. (Dutch Ministry of Housing Spatial Planning and Environment (VROM), 2013).

2. Decree No. 153/2016 Coll. *Vyhláška č. 153/2016 Sb. ze dne 9. května 2016 o stanovení podrobností ochrany kvality zemědělské půdy a o změně vyhlášky č. 13/1994 Sb., kterou se upravují některé podrobnosti ochrany zemědělského půdního fondu (in Czech)*.
